# 2,2′-(Disulfanedi­yl)bis­[4,6-(4-fluoro­phen­yl)pyrimidine]

**DOI:** 10.1107/S1600536812001912

**Published:** 2012-01-21

**Authors:** Richard Betz, Thomas Gerber, Eric Hosten, Serenthimata Samshuddin, Badiadka Narayana, Balladka K. Sarojini

**Affiliations:** a Nelson Mandela Metropolitan University, Summerstrand Campus, Department of Chemistry, University Way, Summerstrand, PO Box 77000, Port Elizabeth 6031, South Africa; b Mangalore University, Department of Studies in Chemistry, Mangalagangotri 574 199, India; c P. A. College of Engineering, Department of Chemistry, Nadupadavu, Mangalore 574 199, India

## Abstract

The title compound, C_32_H_18_F_4_N_4_S_2_, is a disulfide symmetric­ally substituted with two diaza-*meta*-terphenyl groups. In the crystal, the mol­ecule adopts a twisted conformation with a C—S—S—C torsion angle of −91.82 (7)°. One of the 4,6-(4-fluoro­phen­yl)pyrimidine groups is virtually planar, with dihedral angles between the pyrimidine and benzene groups of 4.00 (8) and 5.44 (8)°, wheares the other is non-planar with analogues dihedral angles of 18.69 (8) and 26.60 (8)°. The planar 4,6-(4-fluoro­phen­yl)pyrimidine groups are involved in π–π stacking inter­actions *via* their 4-fluoro­phenyl groups [centroid–centroid distances of 3.8556 (11) and 3.9284 (11) Å] that assemble the mol­ecules into columns extended along the *a* axis. In addition, the structure is stabilized by C—F⋯π [F⋯centroid = 3.4017 (16) Å], C—H⋯F and C—H⋯π inter­actions.

## Related literature

For our work on the synthesis of different derivatives of chalcones, see: Samshuddin *et al.* (2011 ▶); Fun *et al.* (2010 ▶); Jasinski *et al.* (2010 ▶); Baktır *et al.* (2011 ▶). For the graph-set analysis of hydrogen bonds, see: Etter *et al.* (1990 ▶); Bernstein *et al.* (1995 ▶). Metrical parameters of similar compounds were retrieved from the Cambridge Structural Database (Allen, 2002 ▶).
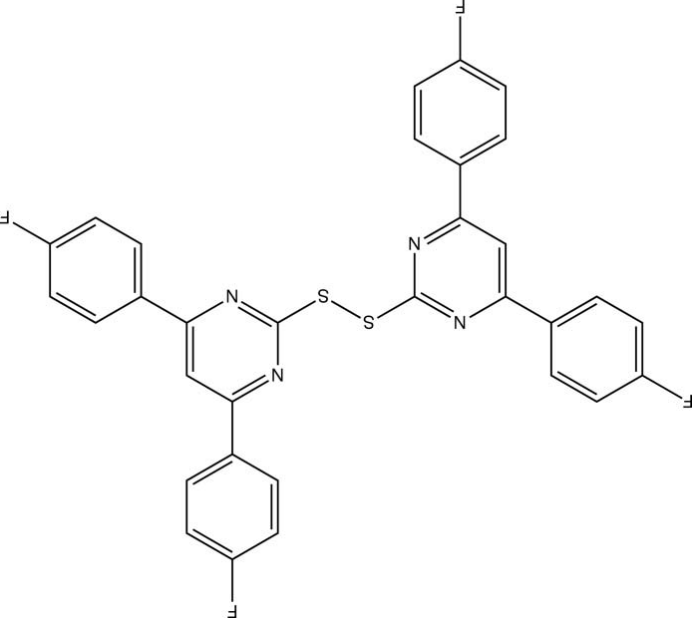


## Experimental

### 

#### Crystal data

C_32_H_18_F_4_N_4_S_2_
*M* *_r_* = 598.62
Triclinic, 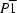

*a* = 9.3371 (2) Å
*b* = 11.3093 (3) Å
*c* = 13.1984 (3) Å
α = 102.364 (1)°
β = 93.094 (1)°
γ = 94.010 (1)°
*V* = 1354.64 (6) Å^3^
*Z* = 2
Mo *K*α radiation
μ = 0.26 mm^−1^
*T* = 200 K
0.50 × 0.42 × 0.29 mm

#### Data collection

Bruker APEXII CCD diffractometer
Absorption correction: multi-scan (*SADABS*; Bruker, 2008 ▶) *T*_min_ = 0.899, *T*_max_ = 1.000
22345 measured reflections
6451 independent reflections
5761 reflections with *I* > 2σ(*I*)
*R*_int_ = 0.019

#### Refinement

*R*[*F* ^2^ > 2σ(*F* ^2^)] = 0.043
*wR*(*F* ^2^) = 0.106
*S* = 1.08
6451 reflections
379 parameters
H-atom parameters constrained
Δρ_max_ = 0.31 e Å^−3^
Δρ_min_ = −0.32 e Å^−3^

### 

Data collection: *APEX2* (Bruker, 2010 ▶); cell refinement: *SAINT* (Bruker, 2010 ▶); data reduction: *SAINT*; program(s) used to solve structure: *SHELXS97* (Sheldrick, 2008 ▶); program(s) used to refine structure: *SHELXL97* (Sheldrick, 2008 ▶); molecular graphics: *ORTEP-3* (Farrugia, 1997 ▶) and *Mercury* (Macrae *et al.*, 2008 ▶); software used to prepare material for publication: *SHELXL97* and *PLATON* (Spek, 2009 ▶).

## Supplementary Material

Crystal structure: contains datablock(s) I, global. DOI: 10.1107/S1600536812001912/gk2426sup1.cif

Supplementary material file. DOI: 10.1107/S1600536812001912/gk2426Isup2.cdx

Structure factors: contains datablock(s) I. DOI: 10.1107/S1600536812001912/gk2426Isup3.hkl

Supplementary material file. DOI: 10.1107/S1600536812001912/gk2426Isup4.cml

Additional supplementary materials:  crystallographic information; 3D view; checkCIF report

## Figures and Tables

**Table 1:** Hydrogen-bond geometry (Å, °) *Cg*2 and *Cg*5 are the centroids of the N3/N4/C5–C8 and C31–C36 rings, respectively.

*D*—H⋯*A*	*D*—H	H⋯*A*	*D*⋯*A*	*D*—H⋯*A*
C33—H33⋯F2^i^	0.95	2.49	3.204 (2)	132
C15—H15⋯*Cg*5^ii^	0.95	2.92	3.751 (2)	147
C23—H23⋯*Cg*2^iii^	0.95	2.98	3.7690 (19)	141

Symmetry codes: (i) 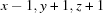; (ii) 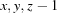; (iii) 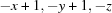.

**Fig. 1.**
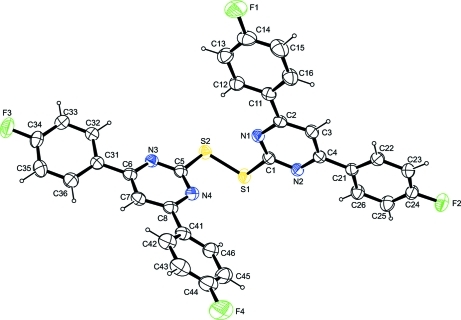
The molecular structure of the title compound, with atom labels and anisotropic displacement ellipsoids drawn at the 50% probability level.

**Fig. 2.**
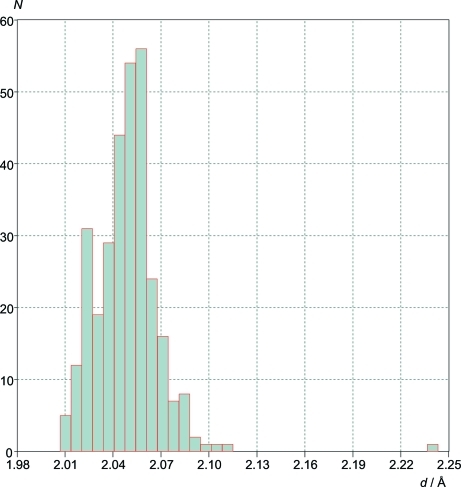
Observed distribution of S–S bond lengths in disulfides featuring sulfur-bonded phenyl-derived aromatic systems (data based on CSD search including all deposited crystal structures up to August 2011).

**Fig. 3.**
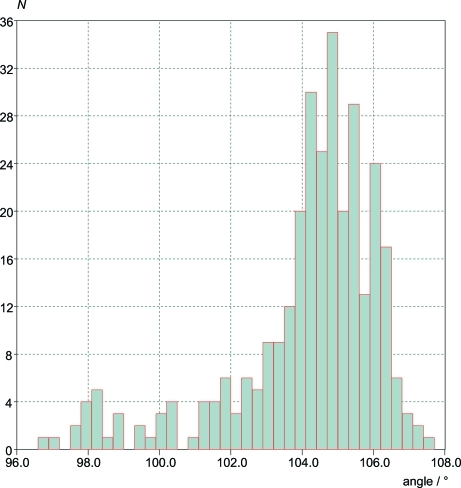
Observed distribution of S–S–Car angles in disulfides featuring sulfur-bonded phenyl-derived aromatic systems (data based on CSD search including all deposited crystal structures up to August 2011).

**Fig. 4.**
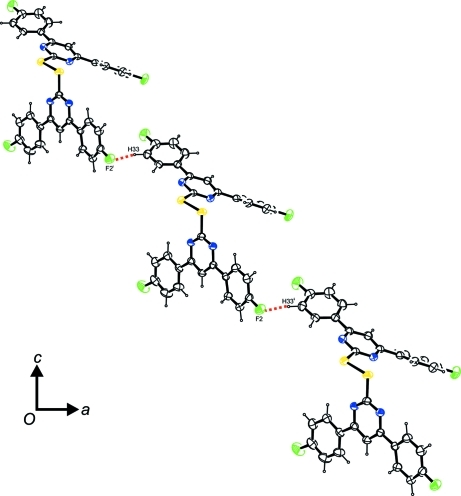
Intermolecular contacts, viewed along [0 1 0]. Symmetry operators: i x-1, y+1, z+1; ii x+1, y-1, z-1.

**Fig. 5.**
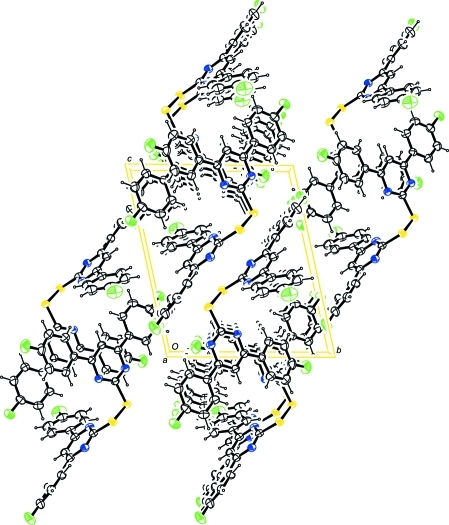
Molecular packing of the title compound, viewed along [-1 0 0] anisotropic displacement ellipsoids drawn at the 50% probability level).

## supplementary crystallographic information

### Comment

In view of the biological importance of pyrimidines and in continuation of our work on the synthesis of various derivatives of 4,4'-difluorochalcone (Samshuddin *et al.*, 2011; Fun *et al.*, 2010; Jasinski *et al.*, 2010; Baktır *et al.*, 2011), we have treated the 4,4'-difluorochalcone with thiourea. Instead of obtaining the expected thio pyrimidine, its dimerization product was obtained whose molecular and crystal structure is reported herein.

The S–S bond length was found at 2.0156 (6) Å and the S–S–C_ar_ bond angles were measured at 104.74 (6)° and 105.41 (5). These metrical parameters are in good agreement with values observed for comparable structures whose crystallographic data has been deposited with the Cambridge Structural Database (Allen, 2002): for 311 comparable structures, a distance range of 2.007–2.237 Å (e.s.d. = 0.021 Å) and an angle range of 96.89–107.54 ° (e.s.d. = 2.1 °) is apparent. The least-squares planes defined by the carbon atoms of the *para*-fluorophenyl groups enclose angles of 4.00 (8)° and 5.44 (8)° as well as 18.69 (8)° and 26.60 (8)°, respectively, with the plane of the aromatic moiety they are bonded to (Fig. 1–3).

In the crystal, C–H···F contacts can be observed whose range falls by more than 0.1 Å below the sum of van der Waals radii of the H and F atoms. These are supported by one of the hydrogen atoms of a *para*-fluorophenyl moiety and connect the molecules to chains along [1 -1 -1]. In terms of graph-set analysis (Etter *et al.*, 1990; Bernstein *et al.*, 1995), these contacts necessitate a *C*(17) descriptor on the unitary level. In addition, there are C–H···π as well as C–F···π interactions (Table 1). The planar 4,6-(4-fluorophenyl)pyrimidine groups are involved in π–π stacking interactions *via* their fluorophenyl groups [centrod-centroid distances of 3.8556 (11) and 3.9284 (11) Å] that assemble the molecules into columns exteded along the *x* axis (Fig. 4).

The packing of the title compound in the crystal structure is shown in Figure 5.

### Experimental

A mixture of 4,4'-difluorochalcone (2.44 g, 0.01 mol) and thiourea (0.76 g, 0.01 mol) was refluxed for 22 h in 25 ml of ethanolic KOH solution. The reaction mixture was cooled to room temperature and kept overnight. The solid product obtained on acidification with acetic acid was filtered and recrystallized from ethanol to obtain a yellow crystalline solid (yield: 51%). Single crystals suitable for the X-ray diffraction study were grown from DMF by slow evaporation at room temperature.

### Refinement

All H atoms were placed in calculated positions (C—H = 0.95 Å) and were included in the refinement in the riding model approximation, with *U_iso_*(H) set to 1.2*U*_eq_(C).

### Crystal data

**Table:**

C_32_H_18_F_4_N_4_S_2_	*Z* = 2
*M**_r_* = 598.62	*F*(000) = 612
Triclinic, *P*1	*D*_x_ = 1.468 Mg m^−^^3^
Hall symbol: -P 1	Melting point: 473 K
*a* = 9.3371 (2) Å	Mo *K*α radiation, λ = 0.71073 Å
*b* = 11.3093 (3) Å	Cell parameters from 9335 reflections
*c* = 13.1984 (3) Å	θ = 2.7–28.3°
α = 102.364 (1)°	µ = 0.26 mm^−^^1^
β = 93.094 (1)°	*T* = 200 K
γ = 94.010 (1)°	Block, colourless
*V* = 1354.64 (6) Å^3^	0.50 × 0.42 × 0.29 mm

### Data collection

**Table:**

Bruker APEXII CCD diffractometer	6451 independent reflections
Radiation source: fine-focus sealed tube	5761 reflections with *I* > 2σ(*I*)
graphite	*R*_int_ = 0.019
φ and ω scans	θ_max_ = 28.0°, θ_min_ = 1.6°
Absorption correction: multi-scan (*SADABS*; Bruker, 2008)	*h* = −12→12
*T*_min_ = 0.899, *T*_max_ = 1.000	*k* = −14→14
22345 measured reflections	*l* = −17→17

### Refinement

**Table:**

Refinement on *F*^2^	Primary atom site location: structure-invariant direct methods
Least-squares matrix: full	Secondary atom site location: difference Fourier map
*R*[*F*^2^ > 2σ(*F*^2^)] = 0.043	Hydrogen site location: inferred from neighbouring sites
*wR*(*F*^2^) = 0.106	H-atom parameters constrained
*S* = 1.08	*w* = 1/[σ^2^(*F*_o_^2^) + (0.0346*P*)^2^ + 0.9028*P*] where *P* = (*F*_o_^2^ + 2*F*_c_^2^)/3
6451 reflections	(Δ/σ)_max_ < 0.001
379 parameters	Δρ_max_ = 0.31 e Å^−^^3^
0 restraints	Δρ_min_ = −0.32 e Å^−^^3^

### Fractional atomic coordinates and isotropic or equivalent isotropic displacement parameters (Å^2^)

**Table:**

	*x*	*y*	*z*	*U*_iso_*/*U*_eq_	
S1	0.37731 (5)	0.31376 (4)	0.26342 (3)	0.03759 (11)	
S2	0.23667 (4)	0.41454 (4)	0.34681 (3)	0.03494 (10)	
F1	−0.06380 (17)	0.81314 (12)	−0.05425 (12)	0.0723 (4)	
F2	0.73521 (13)	−0.01865 (10)	−0.30804 (9)	0.0521 (3)	
F3	0.02419 (15)	1.00874 (11)	0.83195 (11)	0.0637 (3)	
F4	1.10058 (14)	0.77983 (16)	0.33546 (12)	0.0774 (4)	
N1	0.29556 (14)	0.44408 (12)	0.12393 (10)	0.0311 (3)	
N2	0.44257 (14)	0.27892 (12)	0.07397 (10)	0.0320 (3)	
N3	0.26650 (14)	0.61506 (12)	0.48428 (10)	0.0320 (3)	
N4	0.47390 (14)	0.57227 (13)	0.38773 (10)	0.0334 (3)	
C1	0.36867 (17)	0.35313 (14)	0.13960 (11)	0.0309 (3)	
C2	0.29035 (16)	0.46115 (13)	0.02575 (12)	0.0292 (3)	
C3	0.36157 (17)	0.38768 (15)	−0.05031 (12)	0.0334 (3)	
H3	0.3566	0.3989	−0.1197	0.040*	
C4	0.44010 (16)	0.29759 (14)	−0.02361 (11)	0.0294 (3)	
C5	0.34108 (16)	0.54935 (14)	0.41297 (11)	0.0308 (3)	
C6	0.33586 (17)	0.71875 (14)	0.54009 (12)	0.0318 (3)	
C7	0.47672 (17)	0.75261 (15)	0.52079 (12)	0.0349 (3)	
H7	0.5264	0.8257	0.5600	0.042*	
C8	0.54258 (17)	0.67698 (15)	0.44294 (12)	0.0331 (3)	
C11	0.20270 (17)	0.55857 (14)	0.00478 (12)	0.0320 (3)	
C12	0.12508 (18)	0.62160 (15)	0.08269 (14)	0.0370 (3)	
H12	0.1329	0.6046	0.1501	0.044*	
C13	0.0366 (2)	0.70877 (16)	0.06379 (16)	0.0440 (4)	
H13	−0.0162	0.7519	0.1173	0.053*	
C14	0.0272 (2)	0.73108 (16)	−0.03430 (17)	0.0499 (5)	
C15	0.1043 (3)	0.67359 (18)	−0.11258 (16)	0.0552 (5)	
H15	0.0969	0.6925	−0.1793	0.066*	
C16	0.1936 (2)	0.58729 (17)	−0.09272 (14)	0.0459 (4)	
H16	0.2490	0.5473	−0.1460	0.055*	
C21	0.52169 (16)	0.21607 (14)	−0.09826 (12)	0.0300 (3)	
C22	0.53314 (18)	0.23192 (16)	−0.19960 (13)	0.0371 (3)	
H22	0.4902	0.2973	−0.2207	0.045*	
C23	0.60651 (18)	0.15328 (17)	−0.26961 (13)	0.0393 (4)	
H23	0.6139	0.1637	−0.3387	0.047*	
C24	0.66812 (18)	0.06038 (15)	−0.23720 (13)	0.0373 (3)	
C25	0.6638 (2)	0.04285 (16)	−0.13765 (14)	0.0425 (4)	
H25	0.7102	−0.0212	−0.1170	0.051*	
C26	0.58958 (19)	0.12172 (15)	−0.06828 (13)	0.0378 (3)	
H26	0.5848	0.1113	0.0010	0.045*	
C31	0.25489 (17)	0.79344 (14)	0.61996 (12)	0.0327 (3)	
C32	0.10495 (17)	0.77498 (15)	0.61593 (13)	0.0346 (3)	
H32	0.0560	0.7119	0.5637	0.042*	
C33	0.02688 (19)	0.84753 (16)	0.68718 (14)	0.0393 (4)	
H33	−0.0752	0.8360	0.6835	0.047*	
C34	0.1000 (2)	0.93649 (16)	0.76335 (15)	0.0435 (4)	
C35	0.2474 (2)	0.95525 (17)	0.77240 (17)	0.0512 (5)	
H35	0.2954	1.0161	0.8270	0.061*	
C36	0.3244 (2)	0.88330 (17)	0.69998 (15)	0.0460 (4)	
H36	0.4265	0.8954	0.7049	0.055*	
C41	0.69065 (17)	0.70585 (16)	0.41465 (12)	0.0360 (3)	
C42	0.7498 (2)	0.82591 (19)	0.43150 (15)	0.0453 (4)	
H42	0.6955	0.8905	0.4621	0.054*	
C43	0.8882 (2)	0.8507 (2)	0.40345 (16)	0.0530 (5)	
H43	0.9290	0.9321	0.4137	0.064*	
C44	0.9643 (2)	0.7556 (2)	0.36075 (15)	0.0528 (5)	
C45	0.9107 (2)	0.6363 (2)	0.34300 (16)	0.0518 (5)	
H45	0.9668	0.5725	0.3134	0.062*	
C46	0.77154 (19)	0.61228 (18)	0.36986 (14)	0.0421 (4)	
H46	0.7310	0.5306	0.3574	0.050*	

### Atomic displacement parameters (Å^2^)

**Table:**

	*U*^11^	*U*^22^	*U*^33^	*U*^12^	*U*^13^	*U*^23^
S1	0.0500 (2)	0.0384 (2)	0.02800 (19)	0.01296 (17)	0.00568 (16)	0.01157 (15)
S2	0.0364 (2)	0.0365 (2)	0.03120 (19)	−0.00018 (15)	0.00548 (15)	0.00601 (15)
F1	0.0848 (10)	0.0544 (7)	0.0823 (10)	0.0325 (7)	−0.0139 (8)	0.0213 (7)
F2	0.0581 (7)	0.0488 (6)	0.0472 (6)	0.0148 (5)	0.0151 (5)	−0.0004 (5)
F3	0.0668 (8)	0.0465 (7)	0.0701 (8)	0.0144 (6)	0.0177 (6)	−0.0103 (6)
F4	0.0405 (7)	0.1182 (13)	0.0760 (9)	−0.0167 (7)	0.0180 (6)	0.0305 (9)
N1	0.0334 (6)	0.0321 (6)	0.0283 (6)	0.0045 (5)	0.0021 (5)	0.0074 (5)
N2	0.0335 (6)	0.0352 (7)	0.0287 (6)	0.0058 (5)	0.0026 (5)	0.0087 (5)
N3	0.0300 (6)	0.0357 (7)	0.0307 (6)	0.0016 (5)	0.0020 (5)	0.0085 (5)
N4	0.0315 (6)	0.0414 (7)	0.0298 (6)	0.0033 (5)	0.0047 (5)	0.0126 (5)
C1	0.0336 (7)	0.0332 (7)	0.0267 (7)	0.0024 (6)	0.0014 (6)	0.0085 (6)
C2	0.0282 (7)	0.0297 (7)	0.0297 (7)	−0.0012 (5)	−0.0011 (5)	0.0086 (6)
C3	0.0373 (8)	0.0370 (8)	0.0278 (7)	0.0049 (6)	0.0021 (6)	0.0110 (6)
C4	0.0281 (7)	0.0318 (7)	0.0282 (7)	−0.0007 (5)	0.0018 (5)	0.0077 (6)
C5	0.0315 (7)	0.0366 (8)	0.0264 (7)	0.0017 (6)	0.0012 (6)	0.0119 (6)
C6	0.0321 (7)	0.0348 (8)	0.0302 (7)	0.0016 (6)	−0.0001 (6)	0.0115 (6)
C7	0.0336 (8)	0.0393 (8)	0.0321 (8)	−0.0025 (6)	−0.0010 (6)	0.0113 (6)
C8	0.0309 (7)	0.0427 (8)	0.0290 (7)	0.0010 (6)	0.0000 (6)	0.0159 (6)
C11	0.0323 (7)	0.0285 (7)	0.0349 (8)	−0.0006 (6)	−0.0031 (6)	0.0082 (6)
C12	0.0379 (8)	0.0344 (8)	0.0395 (8)	0.0035 (6)	0.0015 (7)	0.0100 (7)
C13	0.0433 (9)	0.0357 (8)	0.0522 (10)	0.0084 (7)	0.0016 (8)	0.0070 (7)
C14	0.0538 (11)	0.0323 (9)	0.0634 (12)	0.0092 (8)	−0.0146 (9)	0.0130 (8)
C15	0.0795 (15)	0.0446 (10)	0.0440 (10)	0.0137 (10)	−0.0101 (10)	0.0165 (8)
C16	0.0620 (12)	0.0413 (9)	0.0364 (9)	0.0113 (8)	0.0000 (8)	0.0117 (7)
C21	0.0273 (7)	0.0323 (7)	0.0291 (7)	−0.0008 (6)	0.0020 (5)	0.0048 (6)
C22	0.0368 (8)	0.0445 (9)	0.0323 (8)	0.0084 (7)	0.0035 (6)	0.0117 (7)
C23	0.0363 (8)	0.0521 (10)	0.0296 (8)	0.0060 (7)	0.0055 (6)	0.0080 (7)
C24	0.0320 (8)	0.0382 (8)	0.0376 (8)	0.0009 (6)	0.0064 (6)	−0.0010 (7)
C25	0.0489 (10)	0.0362 (8)	0.0443 (9)	0.0113 (7)	0.0067 (8)	0.0095 (7)
C26	0.0438 (9)	0.0376 (8)	0.0341 (8)	0.0058 (7)	0.0057 (7)	0.0106 (7)
C31	0.0340 (8)	0.0318 (7)	0.0330 (8)	0.0026 (6)	0.0017 (6)	0.0083 (6)
C32	0.0344 (8)	0.0355 (8)	0.0335 (8)	0.0019 (6)	0.0001 (6)	0.0074 (6)
C33	0.0369 (8)	0.0378 (8)	0.0441 (9)	0.0063 (7)	0.0058 (7)	0.0096 (7)
C34	0.0531 (10)	0.0313 (8)	0.0460 (10)	0.0104 (7)	0.0092 (8)	0.0046 (7)
C35	0.0538 (11)	0.0379 (9)	0.0529 (11)	0.0000 (8)	−0.0012 (9)	−0.0077 (8)
C36	0.0384 (9)	0.0421 (9)	0.0515 (11)	−0.0013 (7)	−0.0011 (8)	−0.0004 (8)
C41	0.0311 (7)	0.0506 (9)	0.0291 (7)	−0.0020 (7)	0.0003 (6)	0.0171 (7)
C42	0.0414 (9)	0.0535 (11)	0.0431 (9)	−0.0045 (8)	0.0022 (7)	0.0183 (8)
C43	0.0474 (10)	0.0652 (13)	0.0479 (11)	−0.0160 (9)	−0.0002 (8)	0.0233 (9)
C44	0.0336 (9)	0.0871 (15)	0.0401 (10)	−0.0092 (9)	0.0050 (7)	0.0229 (10)
C45	0.0390 (9)	0.0746 (14)	0.0452 (10)	0.0052 (9)	0.0113 (8)	0.0184 (10)
C46	0.0360 (8)	0.0555 (10)	0.0373 (9)	0.0002 (7)	0.0069 (7)	0.0161 (8)

### Geometric parameters (Å, °)

**Table:**

S1—C1	1.7827 (15)	C15—H15	0.9500
S1—S2	2.0156 (6)	C16—H16	0.9500
S2—C5	1.7798 (16)	C21—C26	1.395 (2)
F1—C14	1.357 (2)	C21—C22	1.396 (2)
F2—C24	1.3598 (18)	C22—C23	1.384 (2)
F3—C34	1.350 (2)	C22—H22	0.9500
F4—C44	1.357 (2)	C23—C24	1.367 (3)
N1—C1	1.319 (2)	C23—H23	0.9500
N1—C2	1.3492 (19)	C24—C25	1.372 (3)
N2—C1	1.329 (2)	C25—C26	1.386 (2)
N2—C4	1.3482 (19)	C25—H25	0.9500
N3—C5	1.329 (2)	C26—H26	0.9500
N3—C6	1.345 (2)	C31—C36	1.394 (2)
N4—C5	1.324 (2)	C31—C32	1.397 (2)
N4—C8	1.349 (2)	C32—C33	1.384 (2)
C2—C3	1.390 (2)	C32—H32	0.9500
C2—C11	1.481 (2)	C33—C34	1.373 (3)
C3—C4	1.389 (2)	C33—H33	0.9500
C3—H3	0.9500	C34—C35	1.373 (3)
C4—C21	1.480 (2)	C35—C36	1.384 (3)
C6—C7	1.396 (2)	C35—H35	0.9500
C6—C31	1.480 (2)	C36—H36	0.9500
C7—C8	1.389 (2)	C41—C46	1.390 (3)
C7—H7	0.9500	C41—C42	1.397 (3)
C8—C41	1.484 (2)	C42—C43	1.389 (3)
C11—C12	1.389 (2)	C42—H42	0.9500
C11—C16	1.393 (2)	C43—C44	1.368 (3)
C12—C13	1.383 (2)	C43—H43	0.9500
C12—H12	0.9500	C44—C45	1.372 (3)
C13—C14	1.371 (3)	C45—C46	1.389 (2)
C13—H13	0.9500	C45—H45	0.9500
C14—C15	1.367 (3)	C46—H46	0.9500
C15—C16	1.384 (3)		
			
C1—S1—S2	105.41 (5)	C23—C22—C21	120.60 (16)
C5—S2—S1	104.74 (6)	C23—C22—H22	119.7
C1—N1—C2	115.20 (13)	C21—C22—H22	119.7
C1—N2—C4	115.30 (13)	C24—C23—C22	118.61 (15)
C5—N3—C6	115.81 (13)	C24—C23—H23	120.7
C5—N4—C8	114.91 (14)	C22—C23—H23	120.7
N1—C1—N2	129.52 (14)	F2—C24—C23	117.82 (15)
N1—C1—S1	120.58 (12)	F2—C24—C25	119.01 (16)
N2—C1—S1	109.89 (11)	C23—C24—C25	123.17 (15)
N1—C2—C3	120.47 (14)	C24—C25—C26	117.77 (16)
N1—C2—C11	116.29 (13)	C24—C25—H25	121.1
C3—C2—C11	123.22 (14)	C26—C25—H25	121.1
C4—C3—C2	119.16 (14)	C25—C26—C21	121.27 (15)
C4—C3—H3	120.4	C25—C26—H26	119.4
C2—C3—H3	120.4	C21—C26—H26	119.4
N2—C4—C3	120.26 (14)	C36—C31—C32	118.43 (15)
N2—C4—C21	116.35 (13)	C36—C31—C6	121.64 (15)
C3—C4—C21	123.37 (13)	C32—C31—C6	119.93 (14)
N4—C5—N3	129.16 (15)	C33—C32—C31	120.76 (15)
N4—C5—S2	120.13 (12)	C33—C32—H32	119.6
N3—C5—S2	110.71 (11)	C31—C32—H32	119.6
N3—C6—C7	120.32 (15)	C34—C33—C32	118.64 (16)
N3—C6—C31	116.76 (14)	C34—C33—H33	120.7
C7—C6—C31	122.91 (14)	C32—C33—H33	120.7
C8—C7—C6	118.45 (15)	F3—C34—C35	118.55 (17)
C8—C7—H7	120.8	F3—C34—C33	118.85 (17)
C6—C7—H7	120.8	C35—C34—C33	122.60 (17)
N4—C8—C7	121.34 (14)	C34—C35—C36	118.30 (17)
N4—C8—C41	115.90 (15)	C34—C35—H35	120.8
C7—C8—C41	122.76 (15)	C36—C35—H35	120.8
C12—C11—C16	118.72 (15)	C35—C36—C31	121.21 (17)
C12—C11—C2	119.89 (14)	C35—C36—H36	119.4
C16—C11—C2	121.37 (15)	C31—C36—H36	119.4
C13—C12—C11	121.13 (16)	C46—C41—C42	119.34 (16)
C13—C12—H12	119.4	C46—C41—C8	119.66 (16)
C11—C12—H12	119.4	C42—C41—C8	121.00 (16)
C14—C13—C12	118.09 (18)	C43—C42—C41	120.0 (2)
C14—C13—H13	121.0	C43—C42—H42	120.0
C12—C13—H13	121.0	C41—C42—H42	120.0
F1—C14—C15	118.92 (19)	C44—C43—C42	118.61 (19)
F1—C14—C13	118.28 (19)	C44—C43—H43	120.7
C15—C14—C13	122.80 (17)	C42—C43—H43	120.7
C14—C15—C16	118.70 (18)	F4—C44—C43	118.6 (2)
C14—C15—H15	120.7	F4—C44—C45	118.0 (2)
C16—C15—H15	120.7	C43—C44—C45	123.44 (18)
C15—C16—C11	120.50 (18)	C44—C45—C46	117.6 (2)
C15—C16—H16	119.8	C44—C45—H45	121.2
C11—C16—H16	119.8	C46—C45—H45	121.2
C26—C21—C22	118.54 (15)	C45—C46—C41	121.01 (18)
C26—C21—C4	120.41 (14)	C45—C46—H46	119.5
C22—C21—C4	121.05 (14)	C41—C46—H46	119.5
			
C1—S1—S2—C5	−91.82 (7)	N2—C4—C21—C26	3.4 (2)
C2—N1—C1—N2	2.9 (2)	C3—C4—C21—C26	−175.27 (15)
C2—N1—C1—S1	−176.59 (11)	N2—C4—C21—C22	−176.28 (14)
C4—N2—C1—N1	−1.5 (2)	C3—C4—C21—C22	5.1 (2)
C4—N2—C1—S1	178.03 (11)	C26—C21—C22—C23	2.0 (2)
S2—S1—C1—N1	8.39 (14)	C4—C21—C22—C23	−178.31 (15)
S2—S1—C1—N2	−171.23 (10)	C21—C22—C23—C24	−0.4 (3)
C1—N1—C2—C3	−1.5 (2)	C22—C23—C24—F2	177.63 (15)
C1—N1—C2—C11	176.95 (13)	C22—C23—C24—C25	−1.6 (3)
N1—C2—C3—C4	−1.0 (2)	F2—C24—C25—C26	−177.34 (15)
C11—C2—C3—C4	−179.30 (14)	C23—C24—C25—C26	1.9 (3)
C1—N2—C4—C3	−1.3 (2)	C24—C25—C26—C21	−0.2 (3)
C1—N2—C4—C21	179.99 (13)	C22—C21—C26—C25	−1.7 (3)
C2—C3—C4—N2	2.5 (2)	C4—C21—C26—C25	178.59 (15)
C2—C3—C4—C21	−178.93 (14)	N3—C6—C31—C36	162.50 (16)
C8—N4—C5—N3	0.4 (2)	C7—C6—C31—C36	−18.4 (2)
C8—N4—C5—S2	179.94 (11)	N3—C6—C31—C32	−17.8 (2)
C6—N3—C5—N4	−1.1 (2)	C7—C6—C31—C32	161.33 (15)
C6—N3—C5—S2	179.26 (11)	C36—C31—C32—C33	2.6 (2)
S1—S2—C5—N4	10.80 (13)	C6—C31—C32—C33	−177.10 (15)
S1—S2—C5—N3	−169.55 (9)	C31—C32—C33—C34	−1.3 (3)
C5—N3—C6—C7	0.8 (2)	C32—C33—C34—F3	178.93 (16)
C5—N3—C6—C31	179.98 (13)	C32—C33—C34—C35	−0.9 (3)
N3—C6—C7—C8	0.1 (2)	F3—C34—C35—C36	−178.10 (18)
C31—C6—C7—C8	−179.02 (14)	C33—C34—C35—C36	1.7 (3)
C5—N4—C8—C7	0.7 (2)	C34—C35—C36—C31	−0.3 (3)
C5—N4—C8—C41	−178.85 (13)	C32—C31—C36—C35	−1.8 (3)
C6—C7—C8—N4	−0.9 (2)	C6—C31—C36—C35	177.95 (17)
C6—C7—C8—C41	178.62 (14)	N4—C8—C41—C46	−26.2 (2)
N1—C2—C11—C12	−3.7 (2)	C7—C8—C41—C46	154.22 (16)
C3—C2—C11—C12	174.69 (15)	N4—C8—C41—C42	153.20 (15)
N1—C2—C11—C16	177.69 (15)	C7—C8—C41—C42	−26.3 (2)
C3—C2—C11—C16	−3.9 (2)	C46—C41—C42—C43	0.1 (3)
C16—C11—C12—C13	2.0 (3)	C8—C41—C42—C43	−179.39 (16)
C2—C11—C12—C13	−176.68 (15)	C41—C42—C43—C44	−0.8 (3)
C11—C12—C13—C14	0.2 (3)	C42—C43—C44—F4	−178.60 (17)
C12—C13—C14—F1	177.49 (17)	C42—C43—C44—C45	0.7 (3)
C12—C13—C14—C15	−1.9 (3)	F4—C44—C45—C46	179.51 (17)
F1—C14—C15—C16	−178.03 (19)	C43—C44—C45—C46	0.2 (3)
C13—C14—C15—C16	1.4 (3)	C44—C45—C46—C41	−1.0 (3)
C14—C15—C16—C11	0.9 (3)	C42—C41—C46—C45	0.9 (3)
C12—C11—C16—C15	−2.5 (3)	C8—C41—C46—C45	−179.67 (16)
C2—C11—C16—C15	176.11 (17)		

### Hydrogen-bond geometry (Å, °)

**Table:**

Cg2 and Cg5 are the centroids of the N3/N4/C5–C8 and C31–C36 rings, respectively.

**Table:**

*D*—H···*A*	*D*—H	H···*A*	*D*···*A*	*D*—H···*A*
C33—H33···F2^i^	0.95	2.49	3.204 (2)	132
C15—H15···Cg5^ii^	0.95	2.92	3.751 (2)	147
C23—H23···Cg2^iii^	0.95	2.98	3.7690 (19)	141

Symmetry codes: (i) *x*−1, *y*+1, *z*+1; (ii) *x*, *y*, *z*−1; (iii) −*x*+1, −*y*+1, −*z*.
